# Planning priority conservation areas for biodiversity under climate change in topographically complex areas: A case study in Sichuan province, China

**DOI:** 10.1371/journal.pone.0243425

**Published:** 2020-12-23

**Authors:** Yafeng Lu, Pei Xu, Qinwen Li, Yukuan Wang, Cheng Wu

**Affiliations:** 1 Institute of Mountain Hazards and Environment, Chinese Academy of Sciences and Ministry of Water Resources, Chengdu, China; 2 Power China Kunming Engineering Corporation Limited, Kuming, China; Institute of Geographic Sciences and Natural Resources Research Chinese Academy of Sciences, CHINA

## Abstract

Identifying priority conservation areas plays a significant role in conserving biodiversity under climate change, but uncertainties create challenges for conservation planning. To reduce uncertainties in the conservation planning framework, we developed an adaptation index to assess the effect of topographic complexity on species adaptation to climate change, which was incorporated into the conservation framework as conservation costs. Meanwhile, the species distributions were predicted by the Maxent model, and the priority conservation areas were optimized during different periods in Sichuan province by the Marxan model. Our results showed that the effect of topographic complexity was critical for species adaptation, but the adaptation index decreased with the temperature increase. Based on the conservation targets and costs, the distributions of priority conservation areas were mainly concentrated in mountainous areas around the Sichuan Basin where may be robust to the adaptation to climate change. In the future, the distributions of priority conservation areas had no evident changes, accounting for about 26% and 28% of the study areas. Moreover, most species habitats could be conserved in terms of conservation targets in these priority conservation areas. Therefore, our approach could achieve biodiversity conservation goals and be highly practical. More importantly, quantifying the effect of topography also is critical for options for planning conservation areas in response to climate change.

## 1. Introduction

Climate change is widely believed to be the threatening factor of biodiversity, resulting in shifts in distributions, phenology, and behaviors [1–4]. Moreover, the existing conservation system may be ineffective when the species migrated out of nature reserves for adaption climate change. According to the Intergovernmental Panel on Climate Change Fifth Assessment Report, the future climate will change more rapidly, thus the impacts would be magnified consequently [5]. Although expended conservation areas and planning new priority conservation areas have been proposed as a strategy for conservation and management [6], planning results are difficult to accept for implementation due to uncertainties in climate change and their impacts. Currently, developing a comprehensive conservation planning framework to reduce uncertainties may be a significant prerequisite for species conservation under climate change.

As researchers have recognized the urgency to identify conservation areas for adaptation climate change, conservation planning tools have developed from qualitative to quantitative techniques [7]. Linking species distribution models (SDMs) and conservation planning models have shown better performance, such as the Maxent model and Marxan model, while their chances were also taken into account identifying priority conservation areas under climate change [8, 9]. The previous studies suggested that SDMs were the foundation of planning priority conservation areas to figure out where the protection was the most needed [10]. Meanwhile, several studies indicated that the shortcomings in SDMs have been investigated and concerned, leading to inherent uncertainties in identifying priority conservation areas [11, 12]. Due to incomplete knowledge of biodiversity adaptation for climate change, the issues could not fully be eliminated by improving models.

Topographic complexity plays a significant role in biodiversity conservation, which may be a vital factor to reduce uncertainties in planning priority conservation areas [13, 14]. On one hand, topographic complexity supports the diversity of the habitats, which increases the likelihood that the species could disperse suitable habitats with similar climate conditions [15, 16]. Beier suggested that topographic complexity, as an important part of conservation targets, should be conserved in conservation planning [17]. One the other hand, the topographic complexity reduces the human disturbances so that the adaptive capacity of species could enhance under climate change. Williams et al. suggested that the microclimatic buffering of climate change can enhance biodiversity adaptation under climate change [18]. Although previous studies have considered the topographic complexity as well as highlighted the influence of topographic complexity on uncertainties, it is difficult to achieve biodiversity conservation via conserving landscape heterogeneity because species distributions were not entirely consistent with topography diversity.

Given those mentioned issues, this paper aims to develop a framework to plan priority conservation areas for addressing climate change. Specifically, this study will focus on (1) predicting species distributions under climate change; (2) developing an approach to quantify the effect of topographic complexity on adaptation; and (3) incorporating climate change information, biological information, topographic information into planning priority conservation areas to reduce uncertainties.

## 2. Materials and methods

### 2.1. Study area

**Fig 1:**
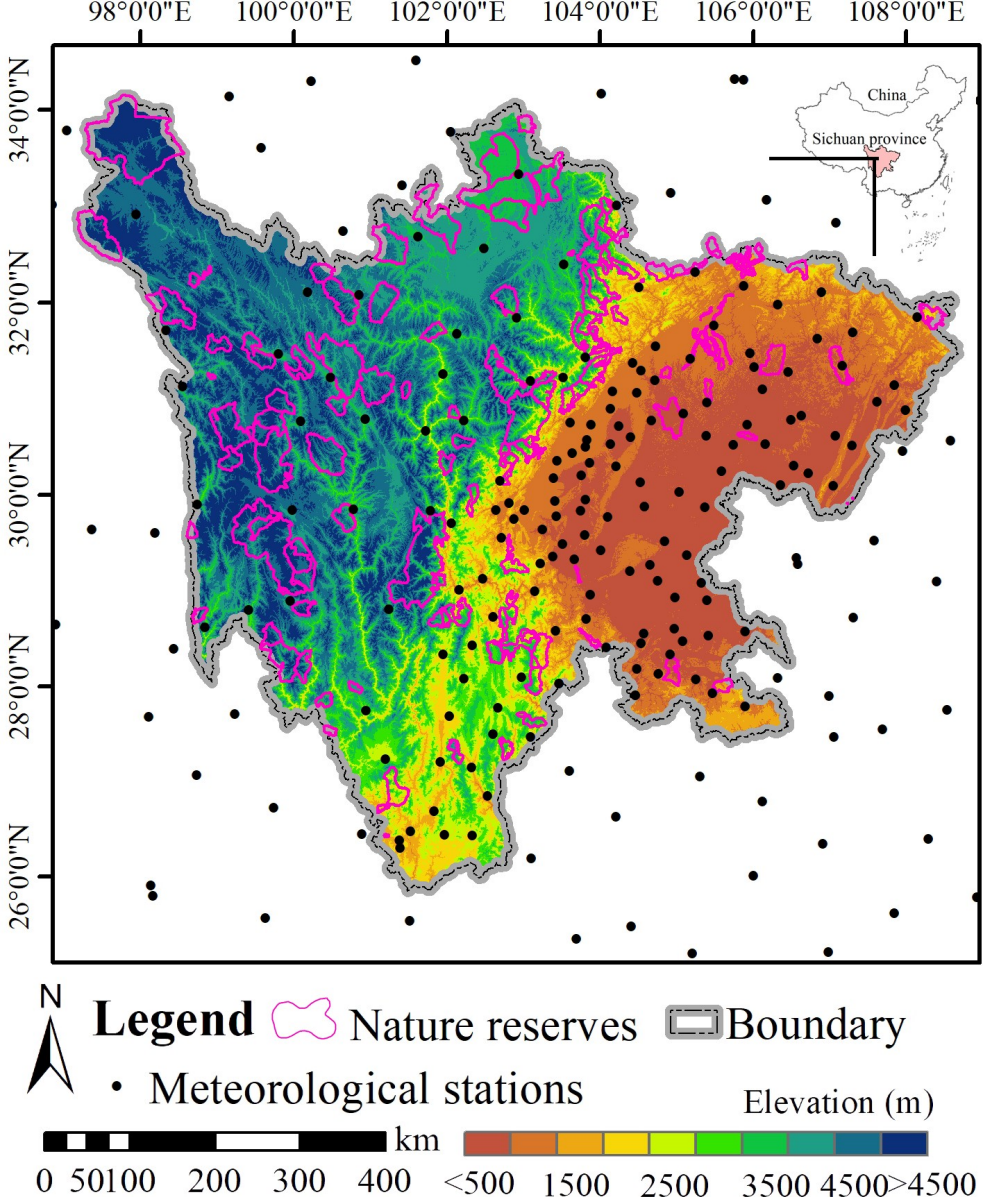
Location of the study area.

Sichuan Province, which is the core area of the Yangtze River as well as one of the top ten land ecological security barriers in southwestern China (Fig 1), is rich in biodiversity [19]. The southwestern area of Sichuan Province belongs to the Hengduan Mountain which is one of the world’s 24 biodiversity hotspots [20]. It has more than 10,000 alpine plant species and 1,200 vertebrate species. Meanwhile, Sichuan’s biodiversity richness has aroused wide attention. Since the 1960s, the Chinese government has established more than 160 nature reserves with a total area of 8.91 million hectares, accounting for 18.3% of Sichuan’s land area. In general, the temperature in the area is decreasing from the east to the west. According to meteorological data, the annual average temperature in Sichuan Province increased by 0.44°C for the period 1961–2010, which has kept increase since 1996. Meanwhile, the Sichuan province is particularly vulnerable to climate extremes, such as droughts, cold temperatures, and heatwaves [21, 22]. However, the habitats of species may be changed due to climate change. These nature reserves could not meet the needs of species protection under climate change, thus offering opportunities to test our methods.

### 2.2. Planning framework

**Fig 2:**
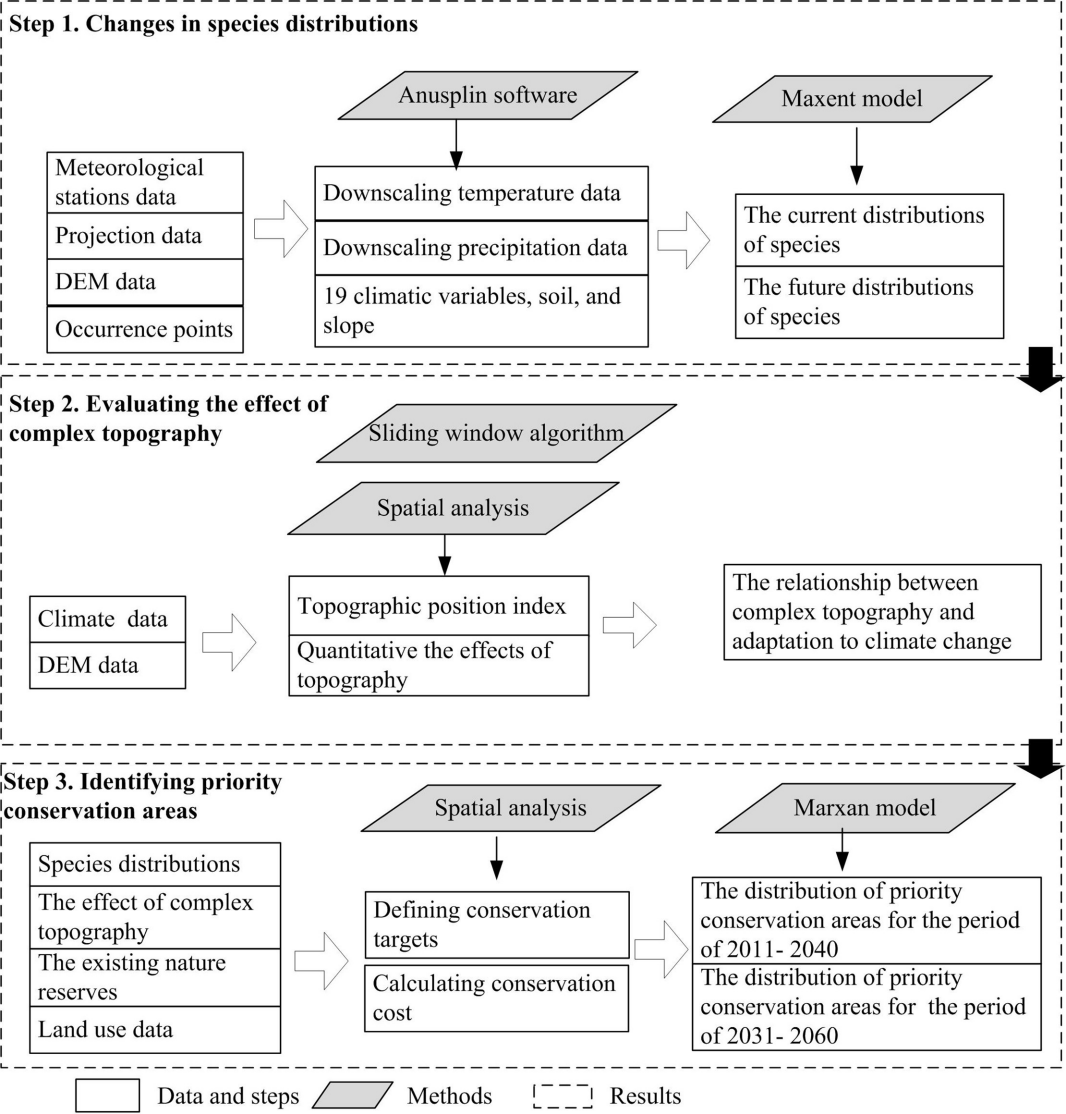
The methodological framework proposed to identify priority conservation areas for climate change.

The conservation planning tools referred to previous studies [23–25]. Spatial distributions of biodiversity were predicted by the Maxent model, as well as we developed an approach to measure the effect of topography on species for adaptation to climate change. Meanwhile, the Marxan model was used to identify priority conservation areas by incorporating species information, topography information, nature reserves, and land use (Fig 2).

### 2.3. Data

#### 2.3.1. Species data

One of the aims was to include as many species as possible to reduce the uncertainties in the conservation planning framework as a whole. Based on a large number of data collected from field survey (species with 30 occurrence records) and expert knowledge, we selected indicator species that are sensitive to climate change, including 118 kinds of indicator species with 49 kinds of wild plants and 69 kinds of wild animals, respectively. The species information was collected from The Nature Conservancy and Institute of forestry research in Sichuan province (S1 Appendix).

#### 2.3.2. Climate change data

Coupled with the Regional Climate Model and Beijing Climate Center's Climate System Model was employed to project monthly temperature and precipitation for the period of 1981–2060 from the Fifth Report of the International Panel for Climate Change. Then, the model output was calibrated by using data from 204 meteorological stations in and around Sichuan Province (Fig 1). Subsequently, the Anusplin software (version 4.3.2) was used to the downscale temperature and precipitation data at a 1 km x 1 km spatial resolution.

#### 2.3.3. Others

Identifying priority conservation areas involves datasets about species occurrence points, climate change data, digital elevation model (DEM), land use, and soil. Besides species information, and climate data, the land use, road, and settlement data were obtained from Landsat 7. The DEM of 90-m resolution was obtained from the United States Geological Survey (https://earthexplorer.usgs.gov/), which was used to generate the slope. Soil dataset was obtained from the Institute of Soil Science, Chinese Academy of Sciences. All spatial data were converted at a 90 m scale to plan priority conservation areas.

### 2.4. Models

#### 2.4.1. Predicting species distributions

Maxent model has been widely used to analyze changes of species distributions under climate change, while it has been suggested to perform best with few species records [25–28]. To characterize the species distributions of the study area, 21 environmental predictors were selected to assess species distributions, including 19 bioclimatic variables [29], the slope, and the soil types. The definitions of bioclimatic variables and calculation models were available online (http://www.worldclim.org/). To avoid over-fitting in the Maxent model, Spearman’s rank correlation coefficient was selected to test the correlation value between variables. When the threshold value of Spearman’s rank correlations were more than 0.75 according to other related studies [9], all highly correlated environmental predictors were removed.

Additionally, we selected 80% data for model training and 20% data for model testing, keeping other values as default. To determine model variables, jackknife analyses were used to reduce the model reliability when omitted. The maxent model ran 1, 000 iterations of the processes or set the value of threshold to 0.00001. Meanwhile, the Receiver Operating Characteristic plot, the Area Under Curve (AUC) values, and the True Skill Statistics (TSS) were used to measure the accuracy of the Maxent model [30, 31]. The greater value of AUC represented the better performer [32]. Maxent was also used to project the distributions of biodiversity for the period of 2011–2040 and 2031–2060 based on future climate data. According to the previous studies [33, 34], there is a range of the value of the potential species distribution, which was divided into 2 groups: unsuitable habitats (0–0.7); suitable habitats (0.7–1.0) for planning conservation priority areas.

#### 2.4.2. Evaluating the effect of topographic complexity

**Fig 3:**
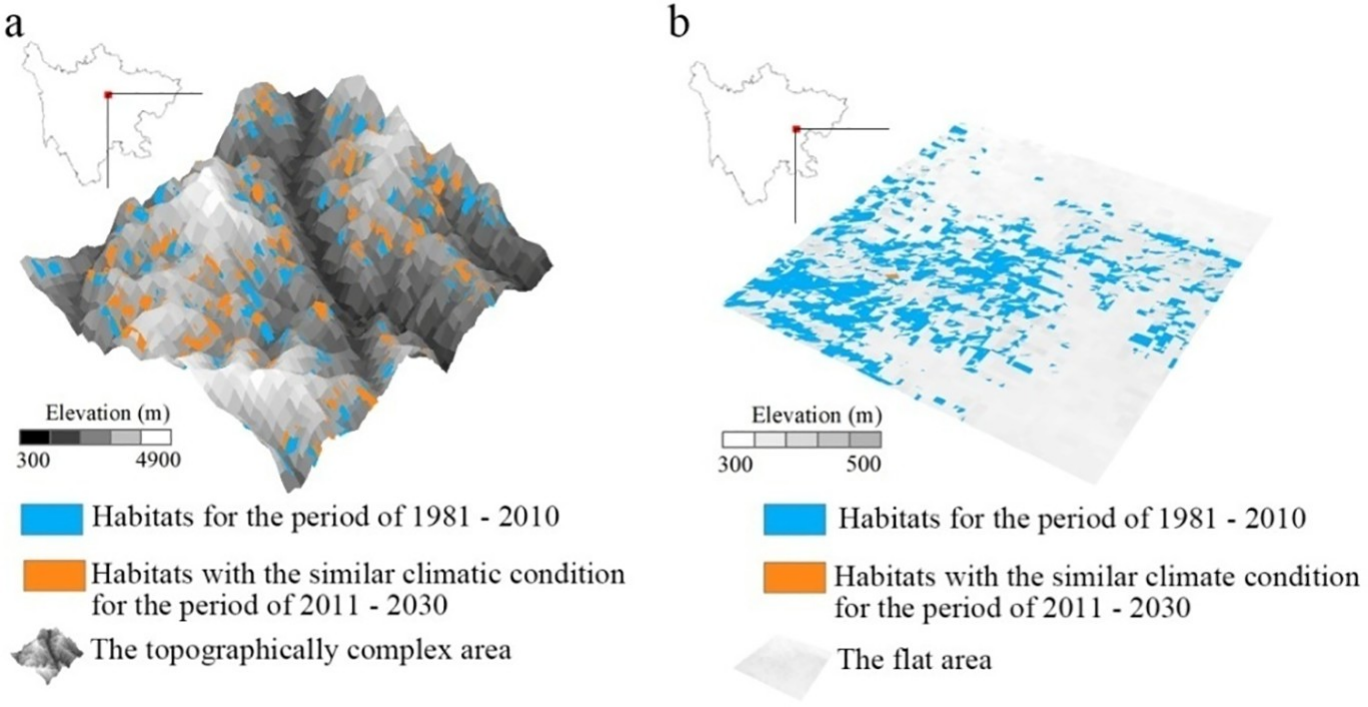
Diagram of the effects of complex topography on climate change adaptation (a represents the topographically complex area, and b represents the flat area).

The species shift to similar climatic conditions for adaptation to climate change, and thus our study rests on two premises to evaluate refugia. First, this study assumed that temperature predominated habitat suitability under climate change. If species could disperse areas with similar temperature conditions, it means that biodiversity could find refugia to adapt. Second, the more topographic complex represents the higher spatial heterogeneity and more types of habitats available, leading to more opportunities for adaptation to climate change in a small area [35]. As shown in Fig 3a and 3b, the species could be greater opportunities to migrate refugia with similar climatic conditions in a small area of topographic complexity than those in a relatively flat area.

According to the above hypothesis, the refugia was defined as the temperatures in the target cell were lower than that in a current species cell due to temperature increase in the future, while Eq (1) was used to assess the adaptation index for each cell based on climatically suitable areas of the surroundings. In calculation, the previous studies suggested that the coldest monthly temperature was a major element to change distributions of species and ecosystems [36], which was selected to assess the effects of topography in our study.

A=∑(Wi/N)/N×100% (1)

In Eq (1), A is the adaptation index, W_i_ is the number of cells of similar climatic conditions in a fixed area, and N is the total number of cells in a fixed area.

To further linking relationships among topographic diversity, adaptation, and climate change, the topographic complexity was divided into 11 types (Fig 5A) by sliding window algorithm [37], while the number of topographic positions was calculated to represent topographic diversity in a fixed area. However, the value of topographic diversity increased with the increase of assessing window area. The mean change-point analysis method could identify the optimal area of the window [38]. Thus, applying the sliding window algorithm and the mean change-point analysis assessed the distributions of topographic diversity for the study area.

#### 2.4.3. Identifying priority conservation areas

Marxan model was developed to determine the spatial distribution of priority conservation areas in terms of the tradeoff between conservation targets and costs. In the model, a minimum-set algorithm was used to achieve planning scenarios on the limitation of conservation costs, which can set different targets based on the number and types of species. The Systematic Conservation Planning tool (Marxan Model v 2.4.3) was applied to plan priority conservation areas for biodiversity in response to climate change in Sichuan province. Meanwhile, we set quantitative targets for conservation features to identify the priority conservation areas for climate change.

The benefit would be the highest in the condition that the protection area accounts for 30% of their habitats [39, 40]. Thus, we set the bottom value of the protection goal to 30% for each species. Meanwhile, the conservation goals of the species were determined by their protection levels. The goals for the percentage of habitat area fall into 3 types: for species belonging to National Protection Grade *I* is 40%, for species belonging to National Protection Grade *II* is 30%, and other species is 30%. Additionally, the uncertainties in climate change associated with future conservation networks change may add additional challenges. To reduce the impacts of uncertainties, the existing nature reserves were also taken as a protection target so that the conservation network could meet the requirements of both current conditions and future scenarios. The goals for the percentage of nature reserves were defined as follows: 90% for national nature reserves, 80% for provincial nature reserves, and 70% for other levels.

Planning priority conservation areas primarily aims to reach the highest ecological benefits from minimum cost. It was desired to avoid in urban, farmland, and road net, while emphasized the effect of topographic complexity. We calculated two different conservation costs in each planning unit. One is a combination of land use and the effect of topography, and the other one is land use only. For combination cost, we set the weight of 1 for two types, as shown in Eq (2). Table 1 displays land use as conservation costs within this study.

$$C=C_{landuse}+(1-A)\times 100$$ (2)

Where, C is a total of conservation cost, C_landuse_ is land use only, and A is the adaptation index (Eq (1)).

**Table 1:** The conservation costs.

Land use type	Cost	Land use	Cost
**Forest**	**5**	**Sand**	**40**
**Shrub**	**5**	**Reservoir**	**50**
**Grass**	**5**	**Barren land**	**50**
**Other forest**	**15**	**Paddy**	**65**
**Lake**	**20**	**Settlements**	**90**
**Marsh**	**30**	**Construction land**	**90**
**Snow**	**30**	**Urban**	**100**
**farmland**	**40**	**Others**	**80**

Additionally, Sichuan province was divided into 25,181 planning units (20 km^2^ each). The conservation species information, nature reserves information and cost information were calculated in each planning unit. For the parameters in the model, we set to run 100 times to calculate the irreplaceability score for each planning unit, which represents the number of counts selected in the optimal solution. Once the score was more than 90, this unit was selected as priority conservation areas. To optimize the spatial distribution, Boundary Length Modifier (BLM) was used to determine optimum value, which was set 0.005 based on the Marxan Good Practice Guide for a compact spatial distribution. Besides the BLM value, other parameters were set the default values in the Marxan model.

## 3. Results

### 3.1. Predicting changes in species distributions

**Fig 4:**
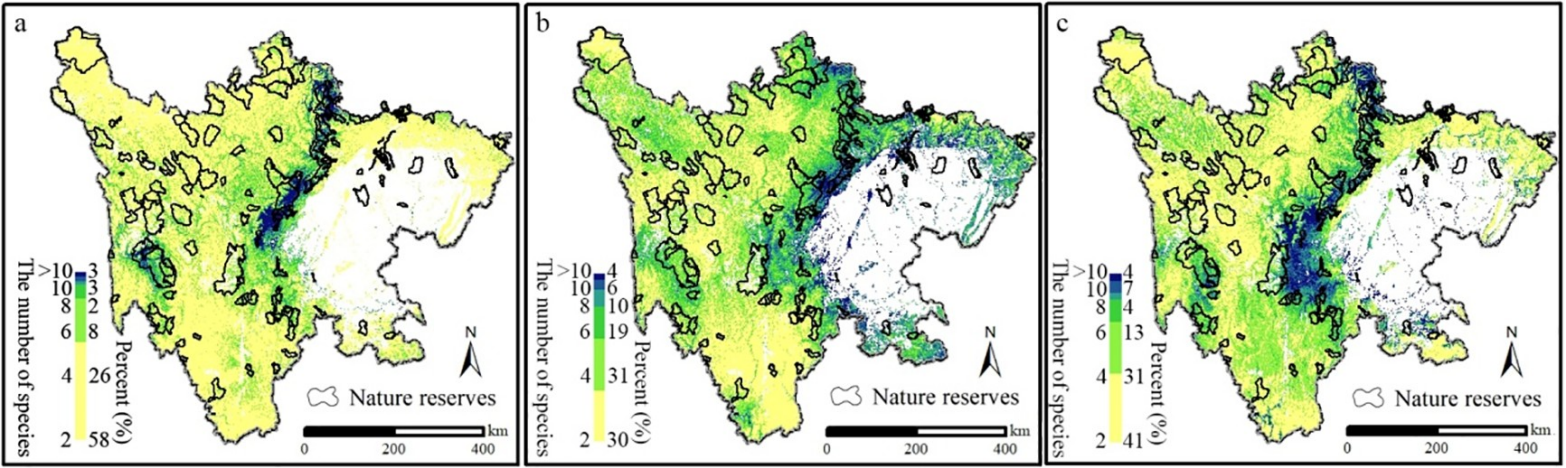
The changes of the number of species in the future (a represents the period of 1981–2010, b represents the period of 2011–2040, c represents the period of 2031–2060).

Fig 4A–4C summarized how the sensitive species distributions would shift based on the IPCC 5 climate model data. After firstly excluding the construction and farmland land, the number of species in each land unit was calculated in three periods. In detail, areas of the highest species richness (equal and more 10) comprised the least area under current climatic conditions (3% of the study area), while mainly distributed in and around Chengdu plain. As climate change, these areas were more abundant (10% and 11% of the study area). Spatially, the areas of biodiversity richness would concentrate on topographic complexity under current and future climatic conditions, which located in the transition from plain to plateau. Additionally, future biodiversity distributions also demonstrated that current nature reserves did not meet conservation. Therefore, climate change would lead to shifts in sensitive species distributions, while planning new conservation areas was a necessity for biodiversity conservation in the study area.

### 3.2. The effect of topography on adaptation to climate change

**Fig 5:**
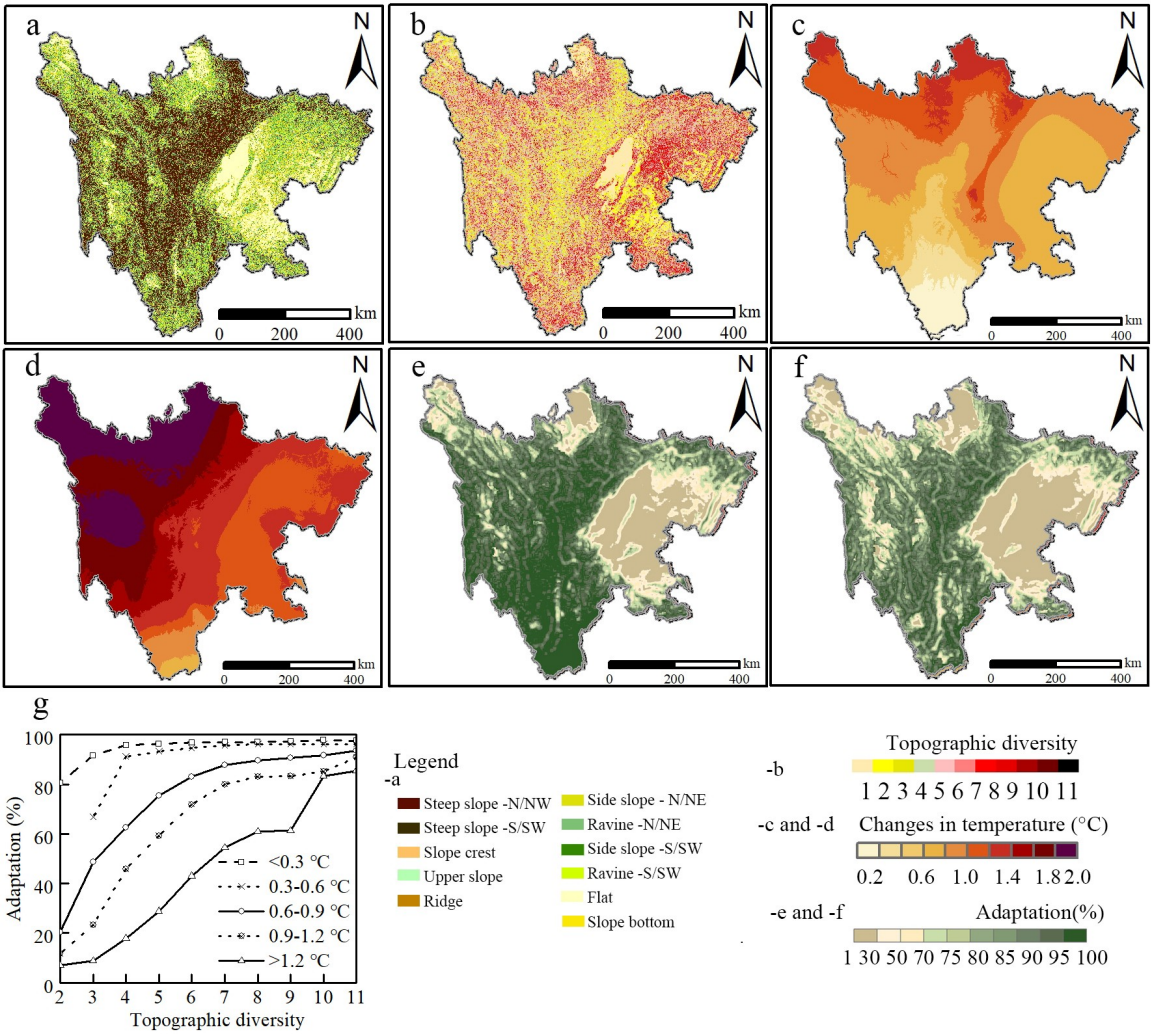
The effect of topography on species adaptation to climate change (a shows topographic position, b illustrates topographic diversity, c and d represent changes in the temperature in the periods of 2011–2040, and 2031–2060, e and f show spatial distributions of adaptation in the periods of 2011–2040, and 2031–2060, and g represents relationships among topographic diversity and the effect of topography).

Fig 5A showed the temporal distribution of topographic position index (TPI). In detail, the area of steep slope comprised the least area and was concentrated in the western part of Sichuan province. Spatially, the areas of side slope were more widely distributed than others. According to TPI, a result from the sliding window algorithm quantitatively assessed an index of topographic diversity, as shown in Fig 5B. Spatially, high diversity areas were mainly located around a transitional zone between the plain and the plateau where the topographic position included 11 types.

Additionally, climate change trends in different periods are shown in Fig 5C and 5D, which shows changes in the coldest monthly temperature in the future. Compared with the period of 1981–2010, the projected climate change shows an obvious increase in Sichuan Province and the increasing range is between 0.2°C- 1.9°C in the future. As for regional comparison, the rise of temperature in the western Sichuan Plateau is higher than that in the Sichuan Basin, indicating that the impact of climate change on biodiversity may be profound in these areas.

Our method was applied to assess the effect of topographic complexity on adaptation to climate change (Fig 5E and 5F), which displayed the spatiotemporal distribution of adaptation for the periods of 2011–2040 and 2031–2060, respectively. The effect decreased with the variability of elevation and from west to east. The highest values of adaptation index near 100% when the coldest monthly temperature increased below 0.3°C. On the contrary, the effect strongly decreased with the temperature reaching values of 1.2°C.

Meanwhile, the results revealed that the relationships among topographic complexity, temperature change, and adaptation could be represented by a nonlinear function (Fig 5G). A significant difference could be observed in the topography effect of different temperature ranges (Wilcoxon signed-rank test, P < 0.05). It also showed that adaptation would decrease to 60% when the coldest monthly temperature rises more than 1.2°C in most regions. Thus, a quantitative evaluation could comprehensively understand the effect of topography on conservation planning.

### 3.3. The distributions of priority conservation areas

**Fig 6:**
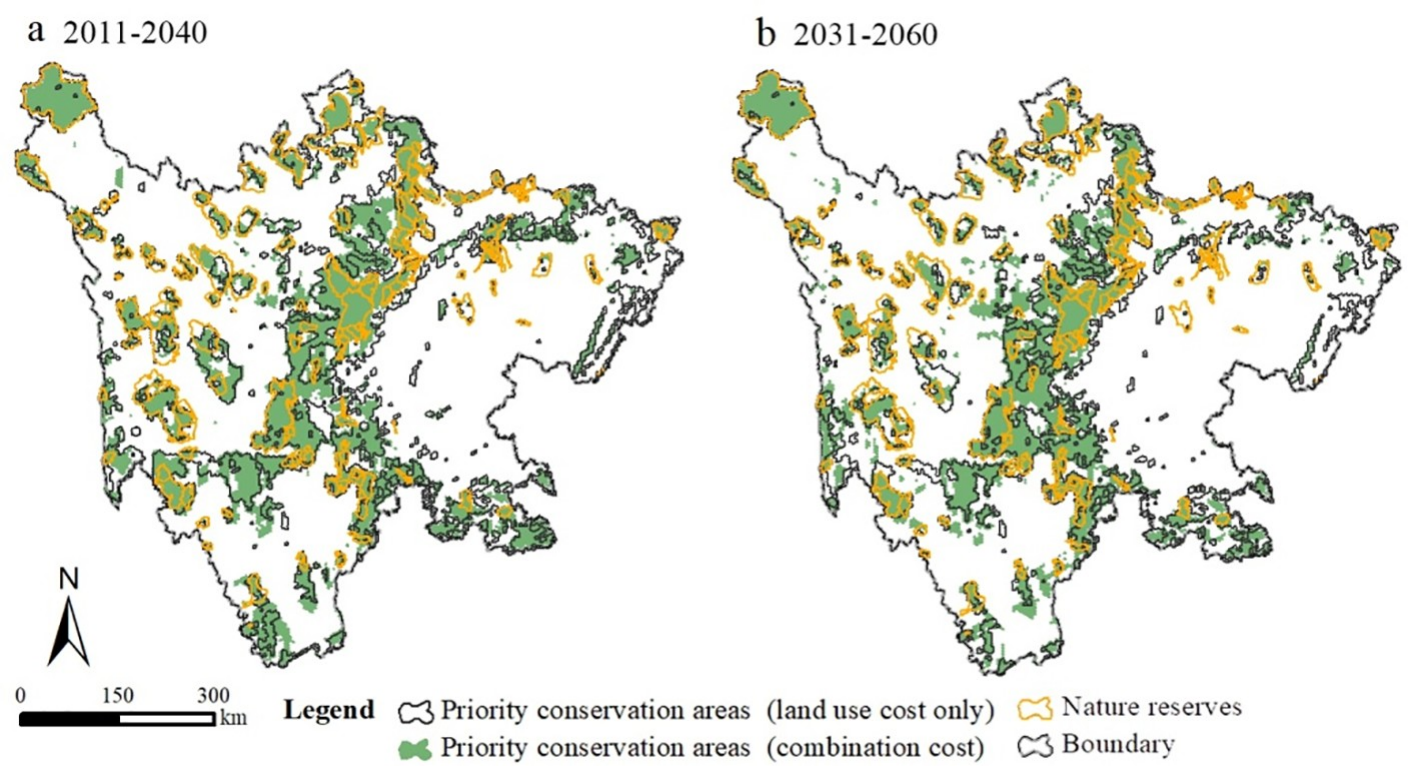
The distributions of priority conservation areas for climate change (a and b represent the priority conservation areas for the period of 2011–2040, and 2031–2060).

The distributions of priority conservation areas for two time periods were optimized by the Marxan model, as shown in Fig 6A and 6B. The area of priority conservation areas covered 136,420 km^2^ (accounting for over 28% of Sichuan province area) and 125,980 km^2^(accounting for over 26% of Sichuan province area), respectively, which were also concentrated in a transitional zone between the plain and the plateau. Compared with current nature reserves (72,408 km^2^), the area of priority conservation areas in this study was nearly 1.8 times larger. To analyze the effect of topography on the conservation framework, different costs were used to optimize priority conservation areas during two periods. The results suggested that there was an obvious spatial mismatch between combination cost and land use-only cost, such as accounting for about 30% of the area of priority conservation areas for 2031–2060. Meanwhile, the area of priority conservation from combination cost is smaller than that from land use-only cost, indicating that conservation cost could be sensitive to conservation planning. Therefore, considering the cost limitation, the results from the combination cost is more practical for biodiversity conservation.

**Fig 7:**
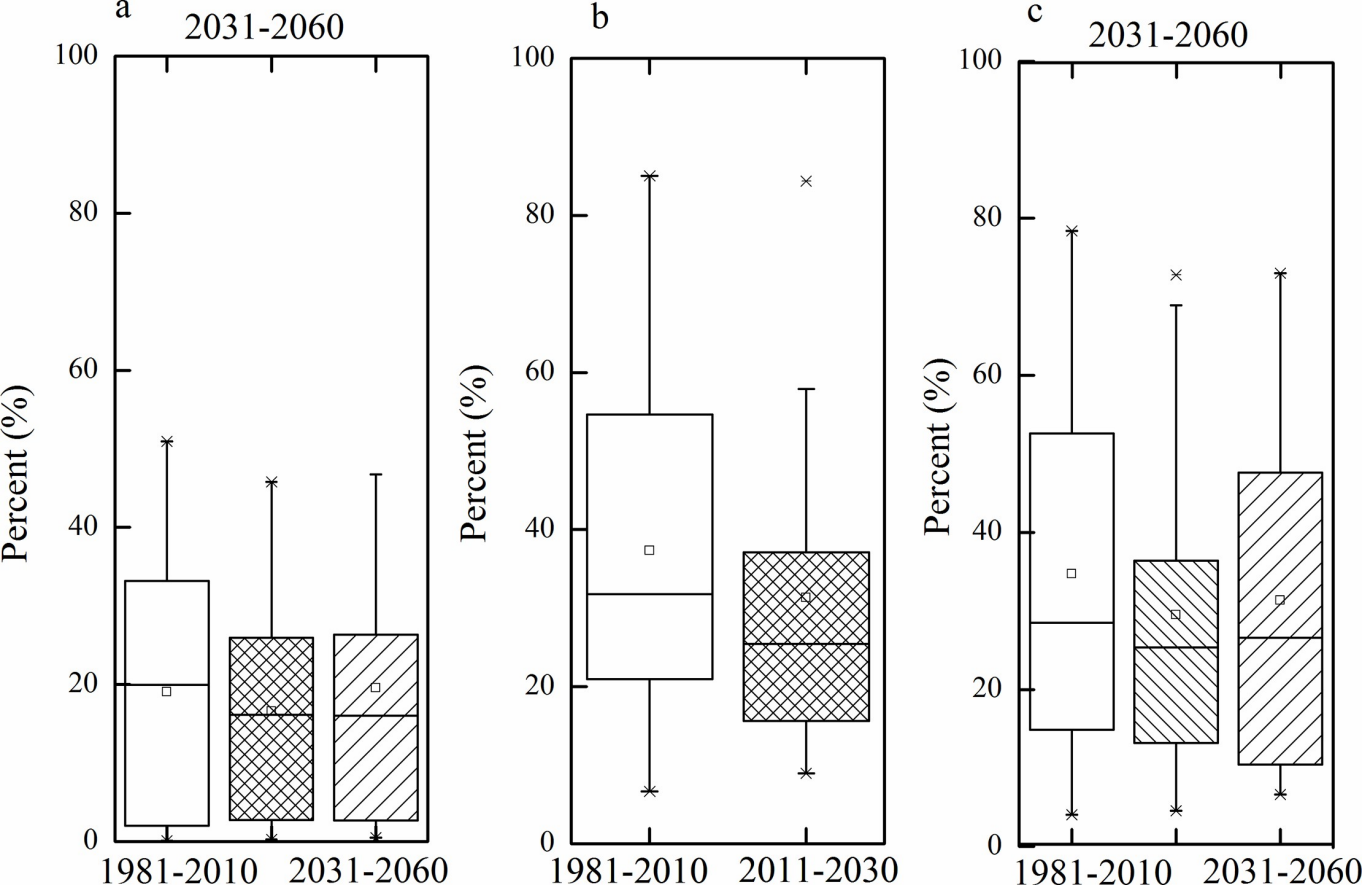
Boxplots showing median, quartiles (25–75%), minimum and maximum of protection area for all national protection species in current nature reserves (a), in priority conservation areas for 2011–2040 (b), and 2031–2060 (c).

To verify the validity of results, box plots revealed protection proportions of species habitats for National Protection Grade *I* and National Protection Grade *II*, as shown in Fig 7A–7C. The results showed that most species could achieve default conservation targets for climate change, indicating that our results are more supportive of biodiversity conservation for climate change.

## 4. Discussion

### 4.1. The impacts of climate change on biodiversity conservation

Evaluating the impact of climate change on biodiversity is the core of planning priority conservation areas. As climate change is a significant driver of species distributions, most sensitive species change their distributions in the future. This is because all suitable areas could not be occupied under current climatic conditions due to biological limitations, including competition, and dispersal [41]. In our results, the changes in climate change may be sufficient to reshape the pattern of species distributions [42–44], while most species would expand their distributions. The previous studies have been pointed out sharply that the distribution of the current conservation network may no longer protect all targeting species, although these reserves were originally planned for climate change [9, 15, 16, 45]. Similar to these studies, our results suggested that current protected areas normally would not maintain all habitats for biodiversity. Thus, identifying larger conservation areas is complementary to enhance adaptation for climate change. Meanwhile, larger conservation areas reduce the impacts of human activities, which can help species in response to adverse environmental conditions.

However, uncertainties in projecting climate change and predicting species distributions are difficult to eliminate. We also found that some species cannot move to suitable climatic conditions for adaptation due to current land use. Besides, there were time lags in the responses of biodiversity to climate change in practice. Despite these limitations, the Maxent model could reveal the impacts of climate change on biodiversity in the study areas as well as provide the estimated species’ persistence within conservation areas [46].

### 4.2. The effect of topographic complexity on adaptation to climate change

As the topographic position index may be a useful element for link habitats and topography, the diversity of topographic position stands for habitats’ richness for adaptation to climate change. First, a topographic complexity could offer a high diversity of climatic conditions, resulting in more possibilities for adaptation [47]. Second, specific topography can change local atmospheric circulation to mitigate climate change [48]. Finally, as for the study area, the field survey showed that the topographic complexity provided shelters for biodiversity in the Quaternary Ice Age, such as the Hengduan Mountains [49, 50].

Although topographic complexity could significantly contribute to enhancing adaptation to climate change, explicit quantification that was required highlighted changes in the effect of topography with temperature. In one such method, the effect of topographic complexity on adaptation was calculated by Rao’s quadratic entropy [51]. Another approach established the relationship between topographic positions and biodiversity to describe the mechanism of adaptation for climate change [52]. Rather than measuring the topographic variation, our approach is different in its focus on quantifying the effect of topography on existing similar climatic conditions in a fixed area. According to our results, topographic complexity in a fixed window can provide more effective shelters for biodiversity below 1.2°C of temperature increase, where the index of topography position diversity is about 6 in Sichuan province. The quantifying results showed that the effect of topographic complexity would decrease or even be ignored with the temperature increase.

Additionally, identifying suitable climatic areas for adaptation to climate change might be useful, which have been used to plan ecological infrastructure for climate change. Nuñez suggested that connecting areas with suitable temperature identified corridors for movement in response to climate change [53]. Anderson demonstrated that new shelters were created in areas with similar topographic characteristics for species under climate change [52]. Similarly, our assumption depends on existing the number of suitable patches in topographically complex areas. Thus, there are good reasons why our approach is well‐suited for quantifying the effect of topographic complexity under climate change.

### 4.3. Conservation planning for climate change

Both species distributions and human activities ultimately determine the pattern of conservation areas [54]. Several approaches have been proposed to predict changes in species distributions to optimize priority conservation areas, our approach highlighted the effect of topography in conservation planning. Incorporating topographic complexity as conservation cost into conservation planning frameworks may reduce uncertainties because it addresses the relationships among biodiversity, topography, climate change, and land use. On one hand, it is noteworthy that the impacts of climate change on species will be more severe for 2011–2040 than those for 2031–2060, yet the area of priority conservation areas show no remarkable change. An explanation is that the effect of topography may provide concentrated refugia for species adaptation. On the other hand, expanded conservation areas may influence local economic development, as well as be criticized as impractical objectives for biodiversity conservation [55]. Our results suggested that planning areas were distributed in topographic complexity where human activities might be inaccessible as well as the agriculture cannot be developed. Thus, as for biodiversity conservation, topographic complexity should be considered explicitly in conservation planning for long–term sustainability.

Furthermore, another significant source of uncertainty is that plan new conservation areas arises from conflicting with current conservation investments. In our study, nature reserves, as important infrastructure for biodiversity conservation, were also planned in new priority conservation areas. According to the distributions of priority conservation areas in different periods, our results are nearly sufficient to cover more than 90% of current protected areas.

As for biodiversity conservation in Sichuan province, managers should enlarge conservation areas as well as make standardized and flexible strategies for conservation networks to reduce uncertainties in the impacts of climate change. First, the distributions of species in transition zones should implement in situ conservation; Second, we recommended that existing nature reserves should reinforce the management while reducing other threats enhance adaptation for climate change in new priority conservation areas. Finally, conservation policies should set management goals of nature reserves for addressing climate change.

## 5. Conclusions

In this study, we developed an algorithm to quantify the effect of topographic complexity on adaptation to climate change, as well as was incorporated into planning conservation areas. Results from quantitative assessment suggested that topographic complexity can be useful to enhance adaptation below 1.2°C of temperature increase. As for Sichuan province, identifying the area of priority conservation areas covered 136,420 km^2^ for 2011–2040, and 125,980 km^2^ for 2031–2060, respectively. Although the conservation areas should be expanded for addressing climate change, planning areas were more realistic and practical due to the effect of topographic complexity. Thus, our approach can be applied to planning priority conservation areas under climate change in mountainous areas, which may reduce uncertainties in the conservation planning framework by quantifying the effect of topographic complexity.

## Supporting information

S1 Appendix The list of indicator species. (DOC)
