# Supplementary material for: Planning priority conservation areas for biodiversity under climate change in topographically complex areas: A case study in Sichuan province, China
Source: PLoS One. 2020 Dec 23;15(12):e0243425. doi: 10.1371/journal.pone.0243425 (PMC7757871; doi:10.1371/journal.pone.0243425)
Supplement: S1 Appendix — (DOC) [file pone.0243425.s001.doc]

S1 Appendix

The list of indicator species

| Species ( Latin name ) | Type | Level |
| --- | --- | --- |
| *Arborophilarufipectus* | Birds | *Ⅰ* |
| *Grusnigricollis* | Birds | *Ⅰ* |
| *Tetraophasis obscurus* | Birds | *Ⅰ* |
| *Tetraophasis obscurus szechenyii* | Birds | *Ⅰ* |
| *Chrysolophus amherstiae* | Birds | *Ⅱ* |
| *Chrysolophus pictus* | Birds | *Ⅱ* |
| *Crossoptilon auritum* | Birds | *Ⅱ* |
| *Crossoptilon crossptilon* | Birds | *Ⅱ* |
| *Ithaginis cruentus* | Birds | *Ⅱ* |
| *Lophura nycthemera* | Birds | *Ⅱ* |
| *Pucrasia macrolopha* | Birds | *Ⅱ* |
| *Syrmaticus reevesii* | Birds | *Ⅱ* |
| *Bambusicolafytchii* | Birds |  |
| *Bonasa sewerzowi* | Birds |  |
| *Corvus macrorhynchos* | Birds |  |
| *Egretta alba* | Birds |  |
| *Garrulaxelliotii* | Birds |  |
| *Lanius schach* | Birds |  |
| *Lerwalerwa* | Birds |  |
| *Myophonus caeruleus* | Birds |  |
| *Pericrocotus ethologus* | Birds |  |
| *Pycnonotus xanthorrhous* | Birds |  |
| *Pyrrhocorax pyrrhocorax* | Birds |  |
| *Rhyacornis fuliginosus* | Birds |  |
| *Tringa totanus* | Birds |  |
| *Zosterops japonicus* | Birds |  |
| *Ailuropoda melanoleuca* | Mammals | *Ⅰ* |
| *Budorcas taxicolor* | Mammals | *Ⅰ* |
| *Cervus albirostris* | Mammals | *Ⅰ* |
| *Cervus nippon* | Mammals | *Ⅰ* |
| *Eozapus setchuanus* | Mammals | *Ⅰ* |
| *Moschus chrysogaster* | Mammals | *Ⅰ* |
| *Panthera uncia(uncia uncia)* | Mammals | *Ⅰ* |
| *Rhinopithecus roxellana* | Mammals | *Ⅰ* |
| *Ailurus fulgens* | Mammals | *Ⅱ* |
| *Capricornis sumatraensis* | Mammals | *Ⅱ* |
| *Cervus unicolor* | Mammals | *Ⅱ* |
| *Lutra lutra* | Mammals | *Ⅱ* |
| *Procapra picticaudata* | Mammals | *Ⅱ* |
| *Pseudois nayaur* | Mammals | *Ⅱ* |
| *Ursus arctos* | Mammals | *Ⅱ* |
| *Ursusthibetanus* | Mammals | *Ⅱ* |
| *Cervus elaphus* | Mammals |  |
| *Elaphodus cephalophus* | Mammals |  |
| *Naemorhedus goral* | Mammals |  |
| *Prionailurus bengalensis* | Mammals |  |
| *Brasenia schreberi* | Plants | *Ⅰ* |
| *Davidia involucrata* | Plants | *Ⅰ* |
| *Kingdonia uniflora* | Plants | *Ⅰ* |
| *Parakmeria omeiensis* | Plants | *Ⅰ* |
| *Cinnamomum camphora* | Plants | *Ⅱ* |
| *Cinnamomum longepaniculatum* | Plants | *Ⅱ* |
| *Fokienia hodginsii* | Plants | *Ⅱ* |
| *Larix mastersiana* | Plants | *Ⅱ* |
| *Meconopsis punicea* | Plants | *Ⅱ* |
| *Picea brachytyla var.complanata* | Plants | *Ⅱ* |
| *Tetracentron sinense* | Plants | *Ⅱ* |
| *Toona ciliata* | Plants | *Ⅱ* |
| *Abies fabri* | Plants |  |
| *Acer catalpifolium* | Plants |  |
| *Alsophila spinulosa* | Plants |  |
| *Betula platyphylla* | Plants |  |
| *Bulbus Fritillaria* | Plants |  |
| *Ceratostigma plumbaginoides* | Plants |  |
| *Cordyceps sinensis* | Plants |  |
| *Cunninghamia lanceolata* | Plants |  |
| *Cupressus chengiana* | Plants |  |
| *Cycas panzhihuaensis* | Plants |  |
| *Dodonaea viscosa* | Plants |  |
| *Euphorbia neriifolia* | Plants |  |
| *Fagus lucida* | Plants |  |
| *Herba Rhodiolae* | Plants |  |
| *Isoetes hypsophila* | Plants |  |
| *Jatropha curcas* | Plants |  |
| *Kobresia setchwanensis* | Plants |  |
| *Magnolia dawsoniana* | Plants |  |
| *Michelia wilsomii* | Plants |  |
| *Opuntia stricta* | Plants |  |
| *Picea likiangensis* | Plants |  |
| *Pinus henryi* | Plants |  |
| *Pinus yunnanensis* | Plants |  |
| *Poa annua* | Plants |  |
| *Populus davidiana* | Plants |  |
| *Pseudotsuga xichangensis* | Plants |  |
| *Quercus aquifolioides* | Plants |  |
| *Quercus baronii* | Plants |  |
| *Quercus pannosa* | Plants |  |
| *Rhododendron przewalskii* | Plants |  |
| *Rhododendron zheguense* | Plants |  |
| *Sabina squamata* | Plants |  |
| *Salix rehderiana* | Plants |  |
| *Sophora davidii* | Plants |  |
| *Taxus mairei* | Plants |  |
| *Tricholoma matsutake* | Plants |  |
| *Tuber mdicum* | Plants |  |
| *Megalobatrachus davidianus* | Reptiles | *Ⅱ* |
| *Tylototriton wenxianensis* | Reptiles | *Ⅱ* |
| *Amolops chunganensis* | Reptiles |  |
| *Amolops loloensis* | Reptiles |  |
| *Amolops mantzorum* | Reptiles |  |
| *Batrachuperus pinchonii* | Reptiles |  |
| *Batrachuperus tibetanus* | Reptiles |  |
| *Bufo gargarizans* | Reptiles |  |
| *Bufo tibetanus* | Reptiles |  |
| *Duttaphrynus melanostictus* | Reptiles |  |
| *Elaphe carinata* | Reptiles |  |
| *Gloydius monticola* | Reptiles |  |
| *Japalura flaviceps* | Reptiles |  |
| *Japalura splendida* | Reptiles |  |
| *Microhyla onata* | Reptiles |  |
| *Nanorana pleskei* | Reptiles |  |
| *Oreolalax major* | Reptiles |  |
| *Ororrana grahami* | Reptiles |  |
| *Ororrana margaretae* | Reptiles |  |
| *Paa boulengeri* | Reptiles |  |
| *Rana limnocharis* | Reptiles |  |
| *Rana nigromaculatta* | Reptiles |  |
| *Zaocys dhumnades* | Reptiles |  |
