# Supplementary material for: Planning priority conservation areas for biodiversity under climate change in topographically complex areas: A case study in Sichuan province, China
Source: PLoS One. 2020 Dec 23;15(12):e0243425. doi: 10.1371/journal.pone.0243425 (PMC7757871; doi:10.1371/journal.pone.0243425)
Supplement: Attachment — Submitted filename: Response to Reviewers.docx [file pone.0243425.s002.docx]

Dear Editors and Reviewers:

Thank you for your letter and the reviewers’ comments concerning the manuscript. All comments are all valuable and very important for revising and improving this study. We have studied comments carefully and have made corrections.

Responds to the reviewer’s comments:

Reviewer #1: The authors attempted to address an important conservation issue linking relationships among topographic diversity, adaptation, climate change, and the complex topography. The authors developed an algorithm to quantify the effect of complex topography on adaptation to climate change, as well as was incorporated into planning conservation areas. These kinds of integrative analyses are very important to identify priority conservation areas considering climate change. The general idea of the study is interesting and deserves attention. However, the manuscript presents, in my opinion, lots of issues that must be attended by the authors in order to improve the performance of the manuscript. Thus, I provide numerous suggestions to increase the clarity of the text. I strongly suggest authors go through a deep and extensive reorganization of the entire manuscript, rewriting paragraphs, removing ambiguities, giving details, adding citations, and making it more clear. Moreover, the figures have typos mistakes and the legend is unclear. Also, although I am not an English native speaker, the manuscript needs a deep check by a native speaker. So, although being unhappy with the actual form and analysis of the manuscript, thumb up for your study. There is lots of work to do yet.

**Thank you for your suggestions, the authors have rewritten paragraphs, added citations, and provided more details in the manuscript.**

Reviewer #2: The manuscript explores the definition of a conservation plan that takes into account both current and future climatic conditions for the area of Sichuan province, while also exploring the effects of topographic complexity and how it may play a major role in buffering climate change effects. The idea is very interesting and the in depth exploration of topographic complexity was novel and promising. Therefore, I believe the paper has its merits and may be suitable for publication if the author made some improvements. I will pinpoint some major topics first and then will proceed to minor questions:

1) First of all, I am not a native English speaker, but I believe the writing needs to be improved and polished in order to reach a publishable level. In several sections I had difficulties in understanding the true meaning of the authors. I highly recommend an overall revision of the writing, as this will definitely improve the level of the manuscript.

**Thank you for your suggestions. The authors have revised the manuscript.**

2) I had some difficulties in understanding a few key concepts in the article. For example, "adaptation to climate change". Adaptation is commonly approached as a property of the species to adapt against unfavorable climatic conditions (by dispersal or micro-evolutionary changes; Foden et al 2013 - PlosOne). Nevertheless, you use it as a property of a given location and create an "Adaptation Index" for each cell based on climatic suitable areas of the surroundings. While I find the method interesting, I think the name could be changed as this Index resembles the idea of a refugia (a concept which you use in your manuscript).

**Thank you for your suggestions, the "Adaptation Index" and the refugia were used in the manuscript.**

3) The methods section needs a more detailed description of the the experiment, especially in the modelling section. There needs to be more detail about the number of occurrences (both total and for each species); the reason of why you chose those species; detail about how you dealt with occurrence of those species outside the province. As for the predictors, is there a link for the Soil database? And there is also the need of more detail about the emission scenarios used within the Regional Circulation Model. There is also the need for a better characterization about the extent (accessible area) used for fitting the models.

**The authors have provided more details in the manuscript. Soil dataset was obtained from theInstitute of Soil Science, Chinese Academy of Sciences.**

4) There is also room for improvement in the modelling procedures. In the introduction you briefly discuss about uncertainty in distribution models but there is a major source of uncertainty not considered in your study: the modelling algorithm (Diniz-Filho et al. (2009). Partitioning and mapping uncertainties in ensembles of forecasts of species turnover under climate change. Ecography, 32(6), 897–906. doi:10.1111/j.1600-0587.2009.06196.x ; Qiao et al (2015). No silver bullets in correlative ecological niche modelling: insights from testing among many potential algorithms for niche estimation. Methods in Ecology and Evolution, 6, 1126–1136. doi:10.1111/2041-210X.12397). You could include other algorithms and perform an ensemble to deal with this uncertainty. This would make your models much more reliable and applicable.Other than that, I also suggest some modifications in the modelling proceddures, such as:-spatial partitioning of the data (instead of random 80-20; Muscarella et al (2014). ENMeval: An R package for conducting spatially independent evaluations and estimating optimal model complexity for MAXENT ecological niche models. Methods in Ecology and Evolution, 5(11), 1198–1205. doi:10.1111/2041-210X.12261)

-Avoid using AUC and replace it with more solid metrics (Barve et al(2011). The crucial role of the accessible area in ecological niche modeling and species distribution modeling. Ecological Modelling, 222(11), 1810–1819. doi:10.1016/j.ecolmodel.2011.02.011; Leroy et al (2018). Without quality presence – absence data , discrimination metrics such as TSS can be misleading measures of model performance. Journal of Biogeography, 45(9), 1994–2002. doi:10.1111/jbi.13402)

**Thank you for your suggestions, the authors have been revised the manuscript.**

**In addition, the authors focus on quantifying the effects of topographic complexity to adaptation rather than improving Maxent model. Uncertainties arise each step in conservation planning for climate change, including climate change projection, species prediction, and conservation areas identification.**

5)Figures are nice, but please consider using colorblind palettes for all figures. There are nice R packages and color palettes within the major GIS softwares (viridis color palette).

Also some suggestions to improve Fig.1 :
-Present a better localization of the location of Sichuan Province within China

-Improve the contrast between the map and the natural reserves, it is very hard to see those.

**Fig 1 has been revised, as follows.**

Some specific points:
-I think the name "topographic complexity" suits better than "complex topography"

**The “topographic complexity” has been used in the manscript.**

L35: Can you fit some example references? I do not know about this distinction.

**The authors have been added references.**

L50: "Although previous studies...". Can you give some examples of such studies?

**The authors have revised, as follows.**

**Williams et al. suggested that the microclimatic buffering of climate change can enhance biodiversity adaptation under climate change [18]. Although previous studies have considered the topographic complexity as well as highlighted the influence of topographic complexity on uncertainties, it is difficult to achieve biodiversity conservation via conserving landscape heterogeneity because species distributions were not entirely consistent with topography diversity.**

L56-59: This could be improved by removing the itemization and presenting those ideas in a sentence.

**The authors have revised, as follows.**

**Given those mentioned issues, this paper aims to develop a framework to plan priority conservation areas for addressing climate change. Specifically, this study will focus on (1) predicting species distributions under climate change; (2) developing an approach to quantify the effect of topographic complexity on adaptation; and (3) incorporating climate change information, biological information, topographic information into planning priority conservation areas to reduce uncertainties.**

L76: It will be more clear if you change the order which sections 2.3 and 2.4 are introduced in the manuscript.

**The authors have changed the order.**

L81: What is the slope factor?

**The slope was generated by a DEM of 90 m resolution. The authors added the slope information.**

L90: (About the 0.7 Threshold) Where did that number came from? If you want to recur to a threshold, I highly recommend using evaluation metrics that rely on Threshold (Jaccard/Sorensen). It is impossible to tell how accurate is the 0.7 threshold using AUC.

**The authors have revised this paragraph, as follow.**

**Additionally, we selected 80% data for model training and 20% data for model testing, keeping other values as default. To determine model variables, jackknife analyses were used to reduce the model reliability when omitted. The maxent model ran 1000 iterations of the processes or set the value of threshold to 0.00001. Meanwhile, the Receiver Operating Characteristic plot, the Area Unde Curve (AUC) values, and the True Skill Statistics (TSS) were used to measure the accuracy of the Maxent model [29, 30]. The greater value of AUC represented the better performer [31]. Maxent was also used to project the distributions of biodiversity for the period of 2011 – 2040 and 2031 – 2060 based on future climate data. According to the previous studies [32, 33], there is a range of the value of the potential species distribution, which was divided into 2 groups: unsuitable habitats (0 - 0.7); suitable habitats (0.7 -1.0) for planning conservation priority areas.**

L109: What is this fixed area?

**The fixed area represents the size of the sliding window algorithm. The value of topographic diversity increased with the increase of assessing window area. Thus, the mean change-point analysis method was used to identify the optimal area of the window**

L111: (About the moving-window) What was the area of the fixed window? This will have a high impact on the results of your Eq.1, as I assume the fixed area is the area of the window, is that correct? I understand the idea behind the mean change-point, but do you think this window could be more related to species dispersal capabilities? In a logic similar to "suitable cells within species' reach". Mean change-point is more related to the spatial configuration of the topography than species actual dispersal, so it could be affected by the definition of your study area. Is this interpretation correct? This area of access could be very different for one of your birds or one of your mammals, for example.

**The mean change-point analysis method was used to identify the optimal area of the window. The topographic diversity value in a fixed area represents climatic diversity, which could offer a high diversity of climatic conditions, resulting in more possibilities for adaptation.**

L183: What do you mean by areas of complex topography here? Is it topographic position (Fig 5a) or Topographic diversity (Fig 5b)? I could not see such a clear visual relation. Can you make more clear how you reached this result ("...areas of biodiversity richness would concentrate on complex topography under current and future climatic conditions, which located in the transition from plain to plateau.")?

**The spatial distributions of biodiversity (Fig 4) were consistent with the topographic diversity (Fig 5 -b).**

L193: (About the highly topographic diverse areas) Are they on the eastern side of the province? Sorry, this is not very clear to me. I think it may be due to the color scale of Fig5b, but it seems to me that the more diverse areas are on the eastern region of the province. Can you try a different color palette that highlights the topographic diversity differences?

**The authors have revised Fig 5 –b, as follows.**

L202-204: Where did the range from 0.3-1.2 degrees came from? Are those based on different emission scenarios from the Regional Circulation Model?

**The range from 0.3°C - 1.2 °C came from climatic surface data. The Anusplin software was used to the downscale temperature data at a 1 km x 1 km spatial resolution. Coupled with the Regional Climate Model and Beijing Climate Center's Climate System Model was employed to project monthly temperature and precipitation for the period of 1981–2060 from the Fifth Report of the International Panel for Climate Change.**

L239: (About increasing the number of areas for conservation) How feasible is this? We have a similar situation in our country, but there is little to zero money for extra investments in conservation. How is the land use in this region?

**Planning priority conservation areas primarily aims to reach the highest ecological benefits from minimum cost. It was desired to avoid in urban, farmland, and road net, while emphasized the effect of topographic complexity. Thus, conservation costs included land use and the effect of topography. The distributions of conservation areas were identified by Marxan model in cost limitation.**

L241: (About species failing to reach suitable areas in the future) I could not see this in your result. This is a very relevant result! I recommend reading the paper (Alagador, D., Cerdeira, J. O., & Araújo, M. B. (2014). Shifting protected areas: scheduling spatial priorities under climate change. Journal of Applied Ecology, 51(3), 703–713. doi:10.1111/1365-2664.12230). There is a very nice and detailed discussion about shifting protected areas, and this paper could gain a lot by this paper and several others mentioned in this article. They also propose a method to take dispersal to new areas into account, which is a very nice addition to your discussion.

**According to the suggestion, the authors have revised the paragraph, as follows.**

**However, uncertainties in projecting climate change and predicting species distributions are also difficult to eliminate. We also found that some species cannot move to suitable climatic conditions for adaptation due to current land use. Besides, there were time lags in the responses of biodiversity to climate change in practice. Despite these limitations, the Maxent model could reveal the impacts of climate change on biodiversity in the study areas as well as provide the estimated species’ persistence within conservation areas [45].**

L250-251"(About the Quaternary refugia) Can you add a reference study for this?

**The relevant references have been added.**

Reviewer #3: Manuscript - “Planning priority conservation areas for biodiversity conservation under climate change in topographically complex areas: A case study in Sichuan province, China” – PlosOne

**This paper is a well done and very interesting study.**

The aim of this study was to to develop an approach to evaluate the effect of complex topography on adaptation climate change, which could be integrated into a conservation planning framework..
The authors used a good and appropriate modelling approach.

I think that this study is suitable for the publication in PlosOne, I have just few specific comments for this manuscript.

Please, see below my specific comments.

Specific comments:

**Lines 16 – 22: More emphasis for your results**

**The authors have revised the manuscript.**

**Identifying priority conservation areas plays a significant role in conserving biodiversity under climate change, but uncertainties create challenges for conservation planning. To reduce uncertainties in the conservation planning framework, we developed an adaptation index to assess the effect of topographic complexity on species adaptation to climate change, which was incorporated into the conservation framework as conservation costs. Meanwhile, the species distributions were predicted by the Maxent model, and the priority conservation areas were optimized during different periods in Sichuan province by the Marxan model. Our results showed that the effect of topographic complexity was critical for species adaptation, but the adaptation index decreased with the temperature increase. Based on the conservation targets and costs, the distributions of priority conservation areas were mainly concentrated in mountainous areas around the Sichuan Basin where may be robust to the adaptation to climate change. In the future, the distributions of priority conservation areas had no evident changes, accounting for about 26% and 28% of the study areas. Moreover, most species habitats could be conserved in terms of conservation targets in these priority conservation areas. Therefore, our approach could achieve biodiversity conservation goals and be highly practical. More importantly, quantifying the effect of topography also is critical for options for planning conservation areas in response to climate change.**

Line 23: To place them in alphabetic order

**The authors have revised the order of keywords.**

Lines 35 – 36: “Linking species distribution models (SDMs)”…you should write in this way and use the acronym “SDMs” from this point onwards

**The authors have revised the manuscript.**

Lines 34 – 53: to expand this part of the manuscript adding more examples and references

**The authors have revised the manuscript.**

Lines 62 – 69: to add climatic information on the study area

**The authors have revised the manuscript as follows.**

**In general, the temperature in the area is decreasing from the east to the west. According to meteorological data, the annual average temperature in Sichuan Province increased by 0.44℃ for the period 1961 - 2010, which has kept increase since 1996. Meanwhile, the Sichuan province is particularly vulnerable to climate extremes, such as droughts, cold temperatures, and heatwaves [21, 22].**

Line 78: I think that you should add some more recent references as examples to support this your sentence: “Maxent model has been widely used to analyze changes of species distributions under climate change”. I would like to suggest:

Raffini, F., et al., (2020). From Nucleotides to Satellite Imagery: Approaches to Identify and Manage the Invasive Pathogen Xylella fastidiosa and Its Insect Vectors in Europe. Sustainability, 12(11), 4508.

Bertolino, S., et al., (2020). Spatially explicit models as tools for implementing effective management strategies for invasive alien mammals. Mammal Review, 50(2), 187-199.

Journé, V., et al., (2020). Correlative climatic niche models predict real and virtual species distributions equally well. Ecology, 101(1), e02912.

Lines 88 - 89: I think that you should add some more recent references as examples to support this your sentence: “Meanwhile, the Receiver Operating Characteristic plot and associated the Area Unde Curve (AUC) values in the Maxent model were produced to measure the accuracy of the Maxent model”. I would like to suggest:

Di Pasquale, G., et al., (2020). Coastal Pine-Oak Glacial Refugia in the Mediterranean Basin: A Biogeographic Approach Based on Charcoal Analysis and Spatial Modelling. Forests, 11(6), 673. Gebrewahid, Y., et al., (2020). Current and future predicting potential areas of Oxytenanthera abyssinica (A. Richard) using MaxEnt model under climate change in Northern Ethiopia. Ecological Processes, 9(1), 6.

**The authors have added these references in the manuscript.**

Lines 88- 89: To use also the True Skill Statistics (TSS) to assess the performance of your models

Allouche, et al., (2006). Assessing the accuracy of species distribution models: prevalence, kappa and the true skill statistic (TSS). Journal of applied ecology, 43(6), 1223-1232.

**The authors have revised the manuscript, as follows.**

**Meanwhile, the Receiver Operating Characteristic plot, the Area Unde Curve (AUC) values, and the True Skill Statistics (TSS) were produced to measure the accuracy of the Maxent model [29, 30]. The greater value of AUC represented the better performer [31].**

Lines 78 – 92: To add all the information regarding the maxent settings used in this study

**The authors have been revised in new manuscript, as follows.**

**Additionally, we selected 80% data for model training and 20% data for model testing, keeping other values as default. To determine model variables, jackknife analyses were used to reduce the model reliability when omitted. The maxent model ran 1000 iterations of the processes or set the value of threshold to 0.00001. Meanwhile, the Receiver Operating Characteristic plot, the Area Unde Curve (AUC) values, and the True Skill Statistics (TSS) were used to measure the accuracy of the Maxent model [29, 30]. The greater value of AUC represented the better performer [31]. Maxent was also used to project the distributions of biodiversity for the period of 2011 – 2040 and 2031 – 2060 based on future climate data. According to the previous studies [32, 33], the threshold value of AUC was set to above 0.7. All the results of the Maxent model in different periods were divided into 2 groups: unsuitable habitat (0 - 0.7); suitable habitat (0.7 -1.0) for planning conservation priority areas.**

Lines 175 – 227: Well written!

**Thank you for your suggestion.**

Lines 289 – 297: To add some conservation recommendations using your study as a example

**The authors have been revised in the manuscript, as follows.**

**As for biodiversity conservation in Sichuan province, managers should enlarge conservation areas as well as make standardized and flexible strategies for conservation networks to reduce uncertainties in the impacts of climate change. First, the distributions of species in transition zones should implement in situ conservation; Second, we recommended that existing nature reserves should reinforce the management while reducing other threats enhance adaptation for climate change in new priority conservation areas. Finally, conservation policies should set management goals of nature reserves for addressing climate change.**
