# Supplementary material for: Planning priority conservation areas for biodiversity under climate change in topographically complex areas: A case study in Sichuan province, China
Source: PLoS One. 2020 Dec 23;15(12):e0243425. doi: 10.1371/journal.pone.0243425 (PMC7757871; doi:10.1371/journal.pone.0243425)
Supplement: Peer review history

# Author comment: Author response to previous submission

**DOI:** 10.1371/journal.pone.0243425.r001

17 Nov 2020

Attachment Submitted filename: Response to Reviewers.docx Click here for additional data file.

# Editor report: Decision Letter 0

**DOI:** 10.1371/journal.pone.0243425.r002

**Authors:** Lyi Mingyang

23 Nov 2020

Planning priority conservation areas for biodiversity under climate change in topographically complex areas: A case study in Sichuan province, China

PONE-D-20-36181

Dear Dr. Xu,

We’re pleased to inform you that your manuscript has been judged scientifically suitable for publication and will be formally accepted for publication once it meets all outstanding technical requirements.

Within one week, you’ll receive an e-mail detailing the required amendments. When these have been addressed, you’ll receive a formal acceptance letter and your manuscript will be scheduled for publication.

An invoice for payment will follow shortly after the formal acceptance. To ensure an efficient process, please log into Editorial Manager at http://www.editorialmanager.com/pone/, click the 'Update My Information' link at the top of the page, and double check that your user information is up-to-date. If you have any billing related questions, please contact our Author Billing department directly at authorbilling@plos.org.

If your institution or institutions have a press office, please notify them about your upcoming paper to help maximize its impact. If they’ll be preparing press materials, please inform our press team as soon as possible -- no later than 48 hours after receiving the formal acceptance. Your manuscript will remain under strict press embargo until 2 pm Eastern Time on the date of publication. For more information, please contact onepress@plos.org.

Kind regards,

Lyi Mingyang, Ph.D.

Academic Editor

PLOS ONE

Additional Editor Comments (optional):

Perfect! You have well addressed all the comments and suggestions provided by the reviewers. Well done! Good luck to your paper!

Reviewers' comments:

# Editor report: Acceptance letter

**DOI:** 10.1371/journal.pone.0243425.r003

**Authors:** Lyi Mingyang

10 Dec 2020

PONE-D-20-36181 

Planning priority conservation areas for biodiversity under climate change in topographically complex areas: A case study in Sichuan province, China 

Dear Dr. Xu:

I'm pleased to inform you that your manuscript has been deemed suitable for publication in PLOS ONE. Congratulations! Your manuscript is now with our production department. 

If your institution or institutions have a press office, please let them know about your upcoming paper now to help maximize its impact. If they'll be preparing press materials, please inform our press team within the next 48 hours. Your manuscript will remain under strict press embargo until 2 pm Eastern Time on the date of publication. For more information please contact onepress@plos.org.

If we can help with anything else, please email us at plosone@plos.org. 

Thank you for submitting your work to PLOS ONE and supporting open access. 

Kind regards, 

PLOS ONE Editorial Office Staff

on behalf of

Professor Lyi Mingyang 

Academic Editor

PLOS ONE
